# Catch that word: interactivity, serendipity and verbal fluency in a word production task

**DOI:** 10.1007/s00426-019-01279-y

**Published:** 2020-01-09

**Authors:** Wendy Ross, Frédéric Vallée-Tourangeau

**Affiliations:** Department of Psychology, Kingston University, Penrhyn Road, Kingston upon Thames, KT1 2EE UK

## Abstract

Problem solving outside of the cognitive psychologist’s lab unfolds in an environment rich with bodily gesture and material artefacts. We examine this meshwork of internal mental resources, embodied actions and environmental affordances through the lens of a word production task with letter tiles. Forty participants took part in the study which contrasted performance in a high interactivity condition (where participants were able to move letter tiles at will), a low interactivity condition (where movements were restrained) and a shuffle condition (where participants could not move the tiles but were allowed to randomly rearrange the array). Participants were also video recorded to facilitate coding of behaviour. While aggregate performance measures revealed a marginal impact of interactivity on performance, when the participants’ behaviour was taken into account, interactivity had a consistent and statistically significant beneficial effect. Detailed, exploratory examination of a subsample of participants informed the formulation of additional hypotheses tested across the full sample: the luckiness of the shuffle in that condition significantly predicted the number of words produced and a more efficient strategy was significantly easier to enact in the high interactivity condition. Additionally, two detailed case studies revealed several moments when accidental changes to the letter tile array offered unplanned words reflecting a serendipitous coagency as well as many moments when environmental chance was ignored. These data and observations indicate that interactivity, serendipity, and internal cognitive resources determine problem-solving performance in this task.

## Introduction

Problem solving outside of strictly controlled laboratory tests unfolds in an environment rich with bodily gesture, material artefacts and other people. Problem solving research attempts to control such a messy backdrop to better isolate the internal cognitive processes which constitute the problem-solving trajectory and posits a linear and disembodied model of problem solving. However, this is being reassessed in several different ways (Beer, 2014; Kirsh, 2009; Steffensen & Vallée-Tourangeau, 2018; Vallée‐Tourangeau, 2014): Cognition is recast as emergent from a dynamic system in which body, environment and person coordinate and in which all, importantly, are essential to understanding the problem solving process. Problem solving in the wild (to adapt Hutchins, 1995) unfolds along an inherently contingent path that is shaped by planned and unplanned opportunities. It can, therefore, be best understood as an activity that takes shape in a dynamic meshwork of resources and processes, configured from internal mental resources, embodied actions and environmental affordances. Disentangling the complex constituent factors in the cognitive process requires different methods to flesh out the conclusions drawn from aggregated performance indices.

Within this meshwork the role of serendipity deserves consideration; that is, the relational nature of luck. Random and accidental moments in problem solving are noticed and enacted by the problem solver. That such moments are important to problem solving in a highly interactive environment is not a new observation and has been referred to anecdotally (Bocanegra, Poletiek, Ftitache, & Clark, 2019; Maglio, Matlock, Raphaely, Chernicky, & Kirsh, 1999; Steffensen, Vallée-Tourangeau, & Vallée-Tourangeau, 2016). Indeed, the idea that problem solving may be augmented by unplanned changes in the environment seems to occur regularly when problem solvers are given an environment that allows for such accidents. For example, Ormerod, MacGregor and Chronicle (2002) invited participants to solve the 8-coin problem using hexagonal coin tokens and write of ‘serendipituously encounter[ing] an external object or event’ (p. 797). In the same way, when Fleck and Weisberg (2013) presented participants working on the triangle of coins with actual coins to build a model of the solution, the researchers noted that some solution hints could be ‘data-driven' (p. 452), that is offered by changes in the physical configuration of the problem (that might or might not been premeditated). This is perhaps most clearly discussed by Seifert, Meyer, Davidson, Patalano and Yaniv (1994) whose opportunistic assimilation theory assesses the characteristics of the ‘prepared mind’ when presented with these unplanned environmental changes. However, a systematic examination of this contingent and emergent phenomenon which assesses the problem solver as part of a coupled system has yet to be undertaken.

## The word production task

The challenge posed by this meshwork is to design a research platform that offers a window onto relatively open-ended creative problem solving that is nonetheless amenable to detailed quantitative and qualitative analyses. Such a platform should help us determine the role of an agent’s internal resources or cognitive capacities and his or her actions as cued by the affordances offered by a dynamic and malleable external environment; it is the dynamic nature of the external environment that may give rise to felicitous serendipities. One such platform is Maglio et al.’s (1999) word production task. Participants are given seven letter tiles and asked to use those letters to generate words which they announce to the experimenter for a short period of time, commonly 3 or  5 min. While closely related to anagram tasks and the game of Scrabble, this task also differs from these: smaller words can be produced and the rate of production rather than the proximity to a right answer or the highest score is measured. This creates an open problem with more flexibility and one which invites a higher granularity of analysis.

Participants’ performance can be observed in two distinct task environments. In a low interactivity environment, where tile movement is constrained or forbidden, performance reflects mental resources alone, while in a high interactivity environment participants can move the tiles as they see fit to support word production. With the low interactivity procedure, the problem solver is decoupled from her immediate environment: she is invited to solve a problem without using her hands to support thinking either through gesture or rearranging the physical elements that configure a model of the problem (such task environments are often the default procedure employed in problem solving research). In other words, problem solving proceeds from mental simulations of possible solutions. In contrast, a high interactivity task environment places no such constraints on her: participants are presented with physical elements of the problem that can be manipulated to arrive at a solution. In such environments, proto solutions unveil new action affordances and guide attention in ways that are simply not possible in low interactivity conditions.

Additionally, Kirsh (2014) used this task to test more explicitly the role of luck in word production. Alongside a low- and high-interactivity task environment he introduced a shuffle condition to isolate the element of chance. The participants were asked to call out as many words as they could in 3 min from a set of seven letters presented on a computer screen. They were asked to do so in three conditions: interactive (when the letters could be rearranged using a mouse), static (the letters could not be moved) and shuffle (one click shuffled the letters randomly). Kirsh found that the shuffle condition produced a significantly higher number of words than either the interactive or the static condition.^1^

## Performance moderators in word production

There are clear theoretical reasons to suppose that interactivity would benefit solvers in a word production task. By extending the mental workspace outside of the head, the internal letter representations are reified and are easily manipulated freeing up working memory and scaffolding participants’ internal resources (Gavurin, 1967; Vallée-Tourangeau & Wrightman, 2010; Webb & Vallée-Tourangeau, 2009). However, the data supporting a scaffolding effect of a high interactivity environment on a word production task are less clear than might be imagined. The only experiment that demonstrates an unequivocal benefit is reported in Fleming and Maglio (2015) where interactivity not only led to an increase in word production but also to rarer words being produced. While Maglio et al. (1999) documented a small overall benefit for interactivity, when this was broken down into the two different letter sets used, interactivity led participants to produce more words with one letter set but fewer words with another. The former was discovered subsequently to be a harder letter set. However, as the set of letters was the between-subjects factor and the participant sample was small (*n* = 10 for each letter set) with no profiling of individual differences (such as verbal fluency), it is unclear to what extent this difference in performance is a true reflection of the benefits or disadvantages of interactivity. Overall, our review of the existing literature suggests three main moderators of performance in this task: internal dispositions, enacted behaviour and environmental affordances.

### Individual differences in verbal fluency

Where individual differences have been profiled, the internal resources of the participants moderate the benefits of interactivity. Vallée-Tourangeau and Wrightman (2010) found that there was a statistically significant benefit for participants categorised in a low verbal fluency group while the benefit for those in the high verbal fluency group was negligible. This mixed story is echoed by Webb and Vallée-Tourangeau (2009) who looked at the benefits of interactivity in children with and without developmental dyslexia. The control group produced marginally more words with their hands when presented with a hard set of letters and the difference with the easy set was indistinguishable. The children with developmental dyslexia (who were characterised by lower working memory score and a lower verbal fluency) benefitted from interactivity with the easy letter set but there was no difference with the hard set. This indicates that interactivity can have a benefit, but that benefit is not universally enacted and is tied to variations in individual difference profiles and letter set difficulty which further points to a more complex relationship than the straightforward additive one normally suggested.

### Individual differences in behaviour

In a footnote to the Maglio et al. (1999, p. 330) paper, we read that roughly one-third of the participants did not, in fact, use their hands or used their hands very briefly despite being at complete liberty to do so and despite this being the key experimental manipulation. That a significant minority of the sample did not consider it worth using their hands to structure their thoughts, requires us to consider to what extent the participants in these conditions could be said to be using interactivity—rather the condition might be more aptly renamed ‘potential for interactivity’. Experiments investigating the role of interactivity in problem solving tightly control the low interactivity condition and participants’ movements are constrained with them often being requested to lay their hands flat on the table. In contrast, there are no controls and, more importantly, rare consideration of exactly how participants recruit resources in a high interactivity condition. If we are to profile the whole system, then the level of interaction and the type of interaction become important, especially if the difference in conditions does not result in differences in the aggregate indices of performance, such as number of words generated.

It is further unclear how much participants’ behaviour differs as a function of their individual differences. It is plausible that those who do not need the help of the tiles recruit them less. Research on expert Tetris players suggests an inverted U-shaped relationship of action and expertise with complementary actions decreasing as expertise increases (Destefano, Lindstedt, & Gray, 2011). In his word production task, Kirsh (2014) also reports a significantly different strategy between the best and the worst performing participants reflected by the number of shuffles. Excess shuffling represented a disadvantage either because the participants who shuffled more were less able to produce words and so chose to shuffle more frequently to break their impasse or because shuffling too much was itself a poor strategy. This indicates that the relationship between condition and performance is also mediated by the behaviour within the conditions. It is not unreasonable to expect a similar relationship in this task.

### Environmental affordances

As there were no reported constraints in the high interactivity condition in Kirsh (2014), this condition actually affords the widest range of potential strategies: it is theoretically possible to shuffle, move the tiles at will or simply leave a static array. In practice, it seems unlikely that participants could have fully used the range of available possibilities of the high interactivity version. Indeed, if we consider the behaviour in the shuffle condition as reported in Kirsh (2014)—the best performing third shuffled once every 3.7 s, the worst performing third once every 1.9 s—the ease with which the shuffle could be executed using the shuffle button is clear and this demonstrates the short time and low cognitive cost of generating environmental hints in the condition. Given the more cumbersome mouse interface, it would be impossible to mimic this strategy with the high interactivity version in the same time as speedily or effectively: This is a function of the affordances of the digital interface which invited participants to interact with the tiles by either pushing a button in the shuffle condition or selecting with a mouse and dragging the tiles into place. In this environment, it possible that shuffling functioned as an epistemic action (Kirsh & Maglio, 1994) and more closely resembled the actions of a Tetris player (who after all does not know the way the tetromino will fall and selects based on what he or she sees). Indeed, Kirsh acknowledges this: ‘the cost in time and mental effort must be sufficiently low that it pays to keep fishing for hints’ (Kirsh, 2014, p. 19). The environmental affordances of the problem space are thus also important in eliciting different behaviours.

## Interactivity and microserendipity

Much of the experimental literature examining problem solving in a situated and movable environment has concentrated on the scaffolding benefits of externalising and reifying representations (e.g., Vallée-Tourangeau, Steffensen, Vallée-Tourangeau, & Sirota, 2016). However, for Maglio et al. (1999) the benefit of high interactivity was in no small part due to the introduction of randomness supporting intelligent behaviour. They theorised that interactivity is beneficial because it allows the solvers to move with less effort through the problem space, and even to jump to new places without being constrained to return to the original configuration. These jumps reflect new, at times unplanned moves (Maglio et al., 1999). This contingent and non-predictable problem solving path has been supported empirically by detailed qualitative analysis (albeit with a so-called insight problem, Steffensen et al., 2016) which indicates that the external environment offers something more than an extension to the mental workspace; rather unplanned changes in the physical appearance of the problem can be pivotal in signposting a solution.

Chance is inert until it is exploited by an agent. No matter how much luck is present in an environment, it only becomes useful when it is used to produce an outcome (Björneborn, 2017). This interaction of environmentally induced chance and human agency is known as serendipity. Serendipity has been described as ‘at the intersection of chance and wisdom’ (Copeland, 2017, p. 1) and the serendipitous process emerges from an interplay of external and internal factors. It is a truly relational concept: It is not enough for the environment to create lucky situations, an individual must take advantage of those situations. Equally, an individual’s internal resources are not the sole locus of cognitive activity—the opportunities thrown up by the environment are equally important. The central concept of serendipity echoes the central thesis of Kirsh (2014) that chance is beneficial to creativity and problem solving but extends it to suggest that the benefits are not indiscriminate and will accrue to those who are most able to exploit it.

Indeed, this relational aspect of serendipity reflects the oft cited aphorism that ‘*le hasard ne favorise que les esprits préparés’* (Pasteur, 1854). The aspect of the prepared mind has been given some attention in problem solving research. Prepared mind in this way can mean one of two things: individual differences that lead someone to be more disposed to take advantage of environmental luck or the experience leading up to the moment of problem solving. The first has been investigated by those who are interested in how much of the relevant stimuli are processed with the conclusions that the tendency to process more irrelevant information may lead to increased creativity (Agnoli, Franchin, Rubaltelli, & Corazza, 2015). The second is closely related to the ides of incubation in problem solving and especially Seifert et al.’s (1995) opportunistic assimilation hypothesis which has also been explored by Ormerod et al. (2002). They hypothesise that failure to solve a problem leads to an impasse and leaves the problem solver particularly primed for external information that will resolve the failure.

Current research casts serendipity as a phenomenon experienced a posteriori (Martin & Quan-Haase, 2016). This reflects the pure definition of serendipity which suggests that it is the noticing of an unanticipated datum that spawns a new, unsought for, solution, explanation or theory (Edward Foster & Ellis, 2014). However, the definition is slippery (Makri & Blandford, 2012) and has been broadened to include both targeted searches which solve a problem via an unexpected route and also untargeted searches which solve immediate problems (Yaqub, 2018). Core to any definition seems to be that it is a problem solved by unanticipated and unplanned means. Using this, then, we can use the framework of serendipity to understand how these unplanned moments are important along the path to a looked-for solution.

We suggest that micro moments of serendipity (microserendipity) can be examined in the laboratory using a word production task. These consist of moments when environmentally generated chance, unplanned and unanticipated by the problem solver, is noticed. This act of noticing triggers a change in the pathway to the problem solution. Microserendipity can only be measured through a fine-grained analysis of video recording of a participant engaging in problem solving with a physical model of the problem.

## The present experiment

The experiment reported here adapted Kirsh’s (2014) procedure and transferred it to an environment which would be more likely to increase the time and cognitive costs of the shuffle condition to assess whether the role of luck extends across environmental presentations. At the same time, moving the procedure to a physical rather than a digital environment afforded a greater range of movements in the high interactivity environment. We predicted that this would change the behaviour of the participants and elicit more moves in the high interactivity condition and fewer shuffles in the shuffle condition reversing the performance outcomes reported in Kirsh (2014). We therefore hypothesised that the high interactivity condition would yield higher performance, followed by the shuffle condition and then the low interactivity condition.

We also profiled the individual differences of the participants thought to be particularly relevant to this task in light of the evidence from Webb and Vallée-Tourangeau, (2009) and Vallée-Tourangeau and Wrightman (2010). Additionally, by filming participants, we were able to undertake a more granular analysis of the strategies employed. This enabled us to disentangle some of the complexities that drove performance in the high interactivity and the shuffle conditions and develop data-grounded exploratory hypotheses. It further enabled us to assess the numbers of participants in the high interactivity condition who choose to move the letter tiles and the manner with which they chose to do so. We hypothesised that while moving the tiles in the high interactivity condition would be beneficial, it would show an inverted U relationship as already noted in Destefano et al. (2011) such that too little or too much movement would be detrimental to performance.

The detailed analysis of the video data provided us with an opportunity to conduct more finely grained group level analyses of behaviour rather than relying on simple aggregate outcome measures to assess strategy. Further, we used detailed case studies to generate new, data-grounded hypotheses which could be tested across the whole data set. As a result we were able to show how efficient engagement with the information offered by the physical environment enhanced word production with interactivity. We were also able to operationalise the luckiness of a letter shuffle, and demonstrate how luckiness enhanced word production. Finally, we offer an even finer-grained analysis of all the letter arrangements generated by two participants to help us map moments of microserendipity. This fine-grained analysis offered us a window onto the complex, dynamic and serendipitous processes inherent to this task.

## Method

### Participants

Forty-two participants (a mixture of undergraduate and post graduate psychology students) took part in the study in return for course credit. Two declined to be filmed and their data were removed from analysis. This left 40 participants (*F *= 32) aged between 19 and 47 (*M* = 25.65, SD = 7.22). All had either had English as a first language or considered themselves fluent speakers of English.

### Design and procedure

The experiment used a repeated measures design with the order of the three experimental conditions counterbalanced across participants. The three conditions were high interactivity (high), low interactivity (low), and shuffle. The participants were invited to call out as many words as they could from a set of seven letters in 5 min. The instructions for the high interactivity conditions read as follows:

Look at the seven letters in front of you. In a 5 min period I would like to you to make as many words using these 7 letters as you think possible. Words need to be at least three letters long and I encourage you to make full use of the range of letters. To help you do so, you can touch and re-arrange the letter tiles in front of you however you wish. You can re-arrange the tiles in any manner you wish. When you think of a word, say it first, then spell it, I will write it down. For example if you think of the word ‘dog’, say it first ‘dog’ and then spell it ‘DOG’. Proper names and acronyms (e.g., IBM) are not acceptable.

In the low interactivity condition participants were asked to not interact with the tiles in any way or to use their hands to gesture or to point. If they did not adhere to these instructions, the experimenter reminded them and requested they hold their hands in their lap. In the shuffle condition, participants were told they could shuffle whenever they liked and as many times as they liked; however, at no other times were they were allowed to move the tiles or gesture as they generated words. Along with the measures of individual differences (described below) the experimental session lasted approximately 35 min.

### Materials

Three sets of letters were created with the same average number of words of similar frequencies produced. These were normed via an online study (*N *= 134) and were selected for similar numbers of words of similar frequencies produced: the three sets of letters were COTFAED, NDRBEOE, and TVAERWI. A valid word was one which only used the letters available and was three or more letters long. It also needed to appear in the SUBTLEX-UK database (van Heuven, Mandera, Keuleers, & Brysbaert, 2014). This assesses word frequencies on the basis of subtitles of British television speakers, converts these to a 7-point logarithmic scale and assigns them a Zipf score based on their frequency. Note that the lower the Zipf Score the less frequently the word occurs in that corpus. We would therefore judge a low Zipf score as reflecting a deeper search space. The number of words in the norming task along with number of letter and the average Zipf score is reported in Table 1.

**Table 1:** Mean values (and standard deviations) for the selected letter sets from the norming study

	*N*	Number of words	Letter count	Zipf Score
COTFAED	41	20.56 (7.32)	3.67 (0.18)	3.67 (0.16)
NDRBEOE	39	20.33 (6.14)	3.80 (0.20)	4.15 (0.23)
TVAERWI	38	19.95 (6.51)	3.78 (0.20)	4.16 (0.18)

The allocation of letter set to experimental condition was counterbalanced across participants. The letter tiles for each set were initially presented in the same manner (as shown above) for all participants.

### Measures of individual differences

#### Verbal fluency

Verbal fluency was measured with an adapted Thurstone Task (Thurstone, 1938). This is the same task used in Vallée-Tourangeau and Wrightman (2010) and in Webb and Vallée-Tourangeau (2009) and, in these studies, predicted performance in some conditions of both tasks. Participants were instructed to write as many words as possible beginning with the letter “S” for 5 min, and then as many words as they could beginning with “C” for 4 min. A participant’s score was the total number of words produced in the 9-min period.

#### Anagram skills

Correlations between Scrabble expertise (on a national rating scale) and anagram skills have been previously reported (Tuffiash, Roring & Ericsson, 2007) so it seems likely individual differences in this regard may moderate performance on the word production task. Indeed, Friedlander and Fine (2016) suggested that cryptic crossword solvers have naturally good anagramming skills which have a strong parallel with the current study given that these solvers tend to deliberately restructure letters. Twelve anagrams were selected from the set used in Webb, Little and Cropper (2018) which were in turn drawn from Novick and Sherman (2003). Each anagram was solvable within one-, two- or three- letter moves with two-letter moves being the most common. The anagrams were presented on Qualtrics. The participants were given 30 s to come up with a solution to each anagram. They were not allowed to use any external aids such as pen and paper.

#### Extraversion and openness to experience

There is limited research on which personality traits correlate most strongly with this propensity to take advantage of luck in the environment in the current serendipity literature, but it has been suggested that extraversion might be (McCay-Peet, Toms, & Kelloway, 2015). We assessed participants along this dimension using the extraversion elements from a revised version of the same scale (the HEXACO-PI-R; Lee & Ashton, 2018). We also profiled the participants on the openness to experience elements of the same scale given the suggestion that this personality trait is related to the processing of irrelevant information (Agnoli et al., 2015; Friedlander & Fine, 2018).

### Performance measures

Performance was measured in three ways. The primary performance measure was the number of valid words produced in each of the three experimental conditions. The frequency score outlined above was one of two secondary performance measures; the other was the length of the word in number of letters.

## Results

The data reported and analysed here have been uploaded to Open Science Framework: https://osf.io/g8jh5/?view_only=1b4bd078cb014d2db980ea1dcee5c855. The long appendices which include second by second detail of the two case study participants summarized at the end of the Results section can also be found at this link.

### Aggregate performance measures

There were slightly more valid words produced in the high interactivity condition with participants producing an average of 18.35 words in 5 min (SD = 8.48); in the shuffle condition, participants produced slightly fewer words (*M *= 17.22, SD = 6.23) and in the low condition they produced the least (*M* = 17.02, SD = 6.59). However, in a repeated measures analysis of variance (ANOVA), the effect of condition on performance was not significant, *F*(2, 78) = 1.94, *p *= . 146 *ŋp*^2^ = .048. Similarly, there were no significant differences in word length between the conditions: the mean word length in the high condition was 3.68 (SD = 0.212), 3.64 (SD = 0.171) in the low condition and 3.62 (SD = 0.205) in the shuffle condition, *F *< 1. The words produced were marginally rarer in the high interactivity condition as indexed with Zipf scores (high: *M *= 4.172, SD = 0.281; low: *M *= 4.254, SD = 2.74, shuffle: *M* = 4.243, SD = 0.234); however, these means did not differ significantly, *F*(2, 78) = 1.54, *p* = .220, *ŋp*^2^ = .038.

### Individual differences

Table 2 reports descriptive statistics for the measures of individual differences, as well as the matrix of correlations for the measures of performance (number of words produced) and scores on the measures of individual differences (*df* = 38 unless noted otherwise). As expected, there was a strong positive correlation between verbal fluency and anagram skills, *r* = .493, *p* = .001; both were significant predictors of performance in all three conditions (lowest *r* = .601, *p* < .001). Measures of extraversion did not correlate with any measures, nor did openness, with the exception of a negative correlation with word production, *r* = .331, *p* = .037, in the high interactivity condition. The direction of this association is a little difficult to interpret given that openness is sometimes associated with higher self-report measures of creativity, and it may be safer to treat the finding with caution.

**Table 2:** Descriptive statistics and correlations among measures of anagram performance, verbal fluency, openness, extraversion and word production performance in the three experimental conditions

	*M*	SD	1	2	3	4	5	6	7
1. Anagram total	8.90	2.73	–						
2. Verbal fluency	88.33	25.15	.493**	–					
3. Openness	50.53	8.56	−.184	−.090	–				
4. Extraversion	52.23	8.49	.133	.074	.345*	–			
5. High interactivity	18.35	8.49	.601**	.717**	− .331*	−.022	–		
6. Low interactivity	17.03	6.59	.679**	.734**	−.223	−.047	.819**	–	
7. Shuffle condition	17.23	6.23	.630**	.745**	−.166	.086	.848**	.796**	–

*p < .05 level (two-tailed)

**p < .001 level (two-tailed)

### Participant behaviour

We further conducted an analysis of participant behaviour within the conditions. For the low interactivity condition, participant behaviour was controlled so variation was limited but in the high interactivity condition, participants were invited to move the tiles as they wished resulting in a wide range of behaviour. In the shuffle condition, while behaviour was controlled between shuffle the number and timings of shuffles was under the participants’ control.

#### Time interacting

In the high interactivity condition, the amount of time participants spent moving the tiles was coded using ELAN (https://tla.mpi.nl/tools/tla-tools/elan/). The total time interacting with the tiles was assessed from when a participant touched a tile to when he or she stopped touching it. As there were many moments when a participant touched a tile but did not move it, this was further split into neutral moves (which did nothing to alter the array) and active moves (which changed the array in some way, either deliberate or random). Active moves were considered a reflection of interactivity.

**Fig. 1:**
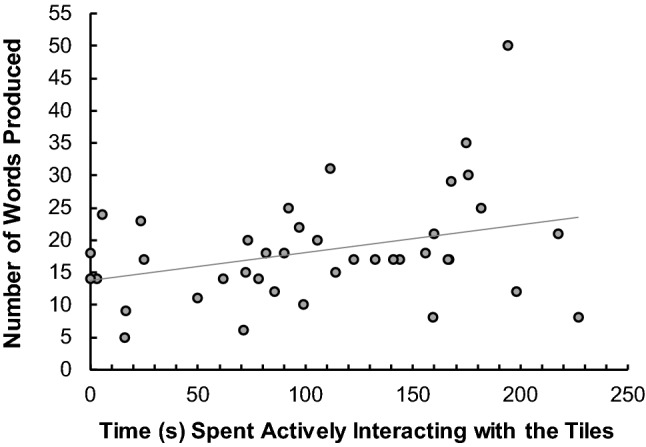
Number of words produced in the high interactivity condition as a function of the time (in s) spent interacting with the letter tiles

The average time spent interacting with the tokens was 106.4 s (SD = 65.1) out of a possible 300 s. Two people chose not to interact at all and from the remaining 38, the shortest amount of time spent moving the tokens was 2.92 s and the longest was 226.9 s. There was a significant correlation between the amount of time spent actively moving the tiles and the number of words produced in the high interactivity condition, *r* = .329, *p *= .038 (see Fig. 1); the correlation was marginally more positive when controlling for fluency, *r*(37) = .356, *p* = .026. This indicates that the amount of time spent interacting had a continually additive effect contrary to our hypothesis. Also contrary to our hypothesis, the relationship between the amount of time spent interacting and the measures of individual differences was not significant, verbal fluency: *r* = .117, *p *= .472; anagram skills: *r* = −.021, *p* = .897. The time spent moving the tiles appears to reflect something beyond individual differences in verbal skills.

To ensure that the total movement time did not reflect an individual difference that would be reflected by an increased performance across all conditions, we examined the correlations between the time spent interacting with the tiles in the high condition with the performance in both the low and shuffle condition. While both were positive, the correlation with words produced in the low condition was not significant, *r* = .219, *p* = .175, nor with words produced in the shuffle condition, *r* = .146, *p* = .365 (and indeed when controlling for fluency these correlations were weaker : low, *r*(37) = .199, *p *= .224 and shuffle, *r*(37) = .093, *p* = .574). This suggests that the time spent moving in the high condition is a unique predictor of the number of words produced in that condition.

#### Shuffling

In the shuffle condition, our hypothesis that the increased time and cognitive cost would lead to a decrease in the number of shuffles from that reported in Kirsh (2014) was upheld by our data. In the shuffle condition the number and timing of the shuffles were also recorded in ELAN. The largest number of shuffles was 3 with a mean of 1.55 (SD = 1.13). Word production performance did not differ as a function of the number of shuffles: 10 participants chose not to shuffle at all (*M* = 18.9, SD = 6.40), 8 shuffled once (*M* = 15.87, SD = 6.12), 12 shuffled twice (*M* = 17.00, SD = 6.50), and 10 shuffled 3 times (*M* = 16.90, SD = 6.43); a one-way between-subjects ANOVA with number of shuffles as a grouping factor revealed that the number of shuffles did not have a significant effect on the number of words produced, *F *< 1. Neither verbal fluency, *r* = .038, *p* = .841 nor anagram performance, *r* = −.011, *p *= .954, correlated with the number of shuffles, supporting the results from the high interactivity condition that suggest that changing the array is not related to those individual differences.

### Exploratory analyses

To our knowledge a detailed analysis of behaviour in this task has not been done before. We first took a subsection of the main sample and subjected these participants to a detailed qualitative analysis of the process of word production to generate further exploratory hypotheses to apply quantitatively to the whole sample. Nine participants were selected for this exploratory analysis. They were selected on the basis of the change in performance from high interactivity to low interactivity: Our goal was to use participants who either benefited the most from the ability to move the tiles to generate words, or those who appeared impeded in their ability to generate words in the high interactivity condition. Thus, this sample of  nine included two participants with the highest boost from interactivity: These participants showed an increase in the number of words produced of 18 and 10, respectively. The two participants who experienced the greatest negative impact of interactivity were also selected. Total word production by these participants declined by 7 and 6, respectively. Three participants who showed no change between the conditions were also selected. Additionally, two participants were selected who also showed behaviour different to the overall trend of data; that is that the more time spent interacting with the tiles the greater the word count. One spent over 3 min (181.9 s) interacting and yet produced 3 fewer words in the high-interactivity condition, the other only spent 5.37 s of the whole 5 min time period interacting with the tiles and yet produced 9 more words in this condition than in the low interactivity. The detailed scrutiny of these nine participants helped us generate three hypotheses which were then tested across the whole sample. The videos of these nine participants were scrutinized for underlying behaviours that may not be captured by the aggregated means and which indicated underlying strategies in approaching the task.

First, we proposed that lucky shuffles led to greater word production. A shuffle leads to a random change in the array and this change may quickly seed new words or obscure them. As serendipity is the enactment of this environmental luck, we can assess this by examining participant behaviour directly after a shuffle. If the shuffle has been useful, it seems likely that the participant would produce a word directly after. If not, then the shuffle has been less useful in breaking the impasse. We therefore indexed luckiness as the time taken to produce a word after a shuffle: the faster a word has been produced, the luckier the shuffle.

Second, higher physical engagement leads to a higher overall word production. Engagement here is a more fine-grained concept then time spent interacting with the tiles. Engagement with the environment can be measured by the responsiveness of the participant to the clues thrown up by it: That is, the more participants respond to the environment, the more words they would produce and, in contrast, the less they use the environment, the more they will be weighed down by the cognitive cost of movement. We termed this measure the efficiency score—that is, the measure of participants’ leverage of environmental opportunities. We expect this to be a greater predictor of word production in the high interactivity environment than in the conditions where this strategy is not as easily enacted.

Third, it seems likely that a word verbalised while the participant was not moving the tokens was more likely to come from internal processes, while one which the participant spoke during movements would reflect changes wrought by the array. We therefore hypothesised that the proportion of words produced while moving the tiles would predict the number of words produced overall in that condition. We next present how these hypotheses fared when evaluated with the data from the whole sample of participants.

#### Luckiness of shuffles

**Fig. 2:**
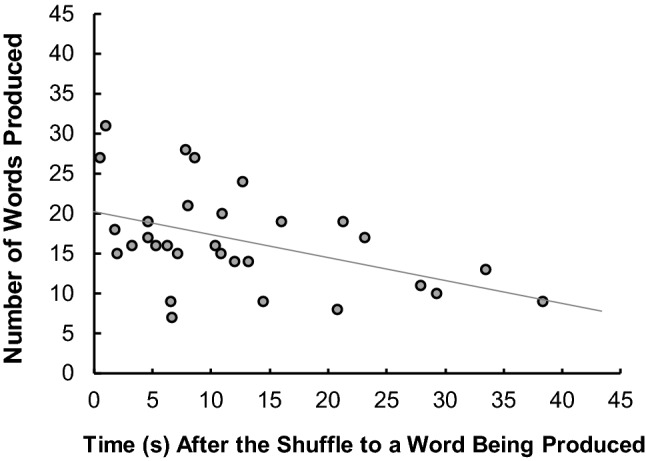
Number of words produced in the shuffle condition as a function of the luckiness of the shuffle

The time from the end of the shuffle to the production of a word was calculated. The end of the shuffle was chosen because participants’ behaviour was once again controlled and their engagement with the tiles limited to internal computations. Luckiness was indexed as the time from the end of the shuffle to the production of a new word. As expected, there was a wide range in times. Indeed, it was possible for a participant to generate a word while relaying the tiles, the change in the letter array presumably triggering an already liminal word. In this case, a negative latency was recorded, which was the time between the participants uttered the word and the time the final tile from the shuffled set was laid on the work surface. Where participants shuffled more than once the average of the times was taken. An analysis of the correlation between the number of words produced in the shuffle condition and the luckiness of the shuffle indexed in this way was conducted on the 30 participants who opted to shuffle (see Fig. 2). This revealed a significant relationship between the number of words produced in that conditions and the luckiness of the shuffle, *r*(28) = −.460, *p* = .011. The shuffle represented an element of nonlinear luck that prompted participants’ performance beyond their individual skills: The relationship between the luck experienced (as operationalised with the average latency to first word produced) did not correlate significantly with verbal fluency *r*(28) = −.316, *p *= .089, or anagram skill, *r*(28) = −.190, *p *= .315.

#### Efficiency score

There are inconsistent benefits to high interactivity. Moving the tiles may not necessarily augment the system’s new word affordances and indeed the additional cost may slow the system down if the benefits are not fully realised. In the word production task where the letters are unchanging, a beneficial strategy is to use the same root and change a single letter. In this way a participant may identify the root -*ate* in the word set and produce the words d-*ate*, f-*ate*, g-*ate*, h-*ate*, l-*ate* and so on depending on the other letters available in the set of seven, a switch between state spaces (Maglio et al. 1999). We hypothesised that an efficient strategy would be easier to follow in a condition where the words are reified physically. Further, we hypothesised it would be a better predictor of performance in the high condition because this strategy could be followed with little working memory cost whereas in a low or shuffle condition the boost from an efficient strategy may be undermined by the cognitive costs required to hold congenial letter arrangements in the head.

We calculated the similarity of the produced word to the word produced immediately before which we call here the efficiency score. This score assumes that when a participant thinks or sees the word, for example, BREAD, it demonstrates a higher efficiency to remove the B and create READ or remove the A and create BRED than to create an entirely new word. Each word generated by a participant was given an efficiency score. Two scores were calculated: the proportion of letters in the same absolute position^2^ and the proportion of letters in the same relative position.^3^ The resulting two proportions give different measures of the similarity of words—it is possible to have words scoring highly in relative position but low in absolute position; for example, if the word READ follows BREAD it scores 0% for absolute position but 100% on the relative position. We therefore used the higher of the two proportions as the efficiency score for each word. Finally, the efficiency scores were averaged across participants.

**Fig. 3:**
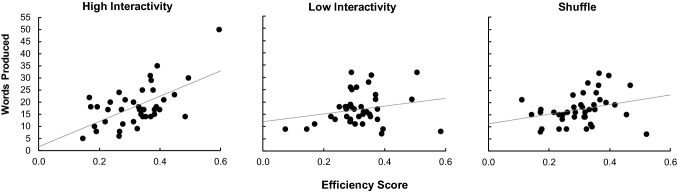
Number of words produced as a function of efficiency score in the high interactivity (left panel), low interactivity (middle panel) and shuffle condition (right panel)

Contrary to our prediction, efficiency scores were similar across all the conditions (high: *M *= .322, SD = .096, low: *M* = .316, SD = .089, shuffle: *M* = 0.303, SD = .090), *F *< 1. This indicates that the participants were using broadly the same strategy across the three conditions. We then examined whether there was an effect of using the strategy on word production in each of the conditions. The relationships are illustrated Fig. 3. The relationship between the efficiency and the total number of words produced in the high interactivity condition was strongly positive, *r* = .597, *p* < .001; however, the level of efficiency was not significantly correlated with word production in either the low, *r* = .223, *p *= .166 or the shuffle condition, *r* = .283, *p* = .076. This indicates that a good strategy is a significant contributor only in the high condition.

### Active words

We further hypothesised that in the high interactivity condition those words produced while the participant was moving, rather than while contemplating the tiles would indicate a higher level of engagement with the environment (having been triggered by ongoing environmental changes) whereas those words produced after movement would be more likely to indicate a word generated from purely internal processes. We therefore calculated the proportion of words announced mid movement and assessed the relationship between this proportion and the number of words produced in the high interactivity condition. A relatively high proportion of words was produced mid movement (*M* = 43.9%, *SD* = 25.7%), however, the relationship between this proportion and the number of words produced was not significant, *r* = .180, *p* = .266 and our hypothesis was not supported.

### Qualitative analysis

Research in interactivity proceeds from the assumption that including the external world in the cognitive ecosystem augments performance but this implies an optional use of the external world; that a problem solver will recruit the environment when she needs it and rely on her own internal processes to solve the problem when they are adequate. Instead, if we suggest that cognition is always systemic then we must consider moments when the external world disadvantages the problem solver. Furthermore, problem solving in a path-rich environment will yield different routes and strategies, the contingent patterns of which may be masked by aggregate data.

We therefore selected a participant who did not benefit from high interactivity (Participant 20; P20) and one who did (Participant 41; P41); P41 had the greatest boost from interactivity. Overall, P41 was higher than average on the measure of verbal fluency: his score was 113 against an average of 88.3 for the sample (+ 0.98 SD). P20 was lower than average on this measure, scoring 52 (− 1.44 SD). Both participants were above average in anagram skill: P41 got all 12 anagrams correct (+ 1.13 SD) and P20 got 10 correct (+ 0.40 SD).

P41 produced 50 words in the high interactivity condition, 32 in the low interactivity condition and 32 in the shuffle condition. He moved the tiles for an above-average time and spent 193.9 s interacting with the tiles, compared to the sample average of 106.4 s (+ 1.34 SD) with over twice as many episodes of activity as average (56 episodes, *M* = 24.72). Indeed, the amount of interaction can be seen in the number of words that were produced during a period of activity. Seventy-three percent of the words generated were produced while moving the tiles. He had a higher than average efficiency score in all conditions although it was higher in the high interactivity condition (high = .60, *M*_high_ = .323, low = .51, *M*_low_ = .316, shuffle = .40, *M*_shuffle_ = .303).

P20 produced 12 words in the low-interactivity environment and 6 in the high-interactivity environment (9 in the shuffle). This indicates that for this problem solver the extended ecosystem was not an aid to thinking, rather it acted as a hinderance to the whole system. She spent less time than average interacting with the tiles, 71.23 s (− .541 SD) However, more of her words were produced during a period of activity (88%). She had a lower than average efficiency score in the high (.26) and low (.29) but higher than average in the shuffle (.32).

The following analyses contrast how the singular trajectory unfolded for each participant and specifically assess the coordination of the different systemic elements. The unit of analysis here is not reduced to the individual problem solver but instead contrasts the problem-solving systems formed by the problem solver, his or her environment and the unfolding of the problem over time. In the analysis that follows words produced by the participant are identified with capital letters (e.g., BODE), possible words not produced are written in lower case bold (e.g., **bed**). Each second of the video is presented in the appendix (found here: https://osf.io/g8jh5/?view_only=1b4bd078cb014d2db980ea1dcee5c855) and the most salient events are highlighted here coded with E and the second to which they refer.

### Inconsistent effects of reification

After saying the first word (not a valid because of the spelling), P20 changed the array to reflect her suggestion. This move was of low utility—the word has already been generated and changing the array did not yield any additional information. Indeed, it is likely to be an impediment to new words because the suggested word is now treated as a unit, potentially blocking new ideas. This was a common strategy for P20 who spelt out every word after saying it indicating that the direction of cognition was internal to external. Changing the array in this way did not yield any benefit and was only a time and cognitive cost as well as perhaps stifling the generation of new combinations.

On the other hand, although P41 reifies his announcements, he does not leave them to stagnate. He breaks the initial set up quickly, creating a circular arrangement (E5), once the word BOD (E6) is identified this leads quickly to BODE (E9) then BORE (E14), BORED (E17) and BORN (E22) before hazarding a guess at BORNE (E28). This pattern is repeated several times (e.g., ROB [E98] to ROBE [E102] to ROD [E106] to RODE [E110]). This pattern of movements would yield a high efficiency score and would be supported by the high interactivity environment because the congenial collections of letters can be reified into a new candidate word. It is noticeable that this appears to only be a useful strategy if the letter tiles are moved sufficiently such as to defeat the anchoring paralysis of a static array. Thus, a level of disengagement is also necessary. Indeed, we can see in E52 the word **bed** is created by the left-over tiles but the participant rather incorporates unused letters to keep the array dynamic so this candidate word opportunity is not banked in; rather, he creates BEND (E54).

### Microserendipity

**Fig. 4:**
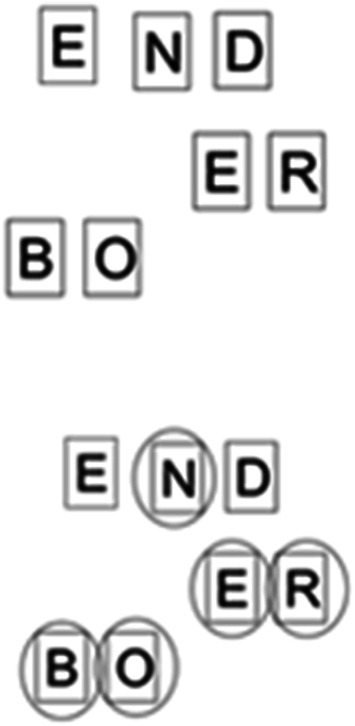
A word discovered through serendipity

The changes to the array yield other left-over collections of letters which are not produced intentionally but are rather an artefact of the movements which have come before. For example, creating the word BODE leaves the letters NRE (E10), later the R and the E become rearranged as artefacts to become ER (E51) which is a much more useful digraph. This movement is not intentional but rather a necessary outcome of a constantly shifting array. For P41, there are several moments when such unplanned moments prompt the following word, which are better understood as moments of microserendipity. The word BRO (E34) leaves the digraph EE on the array. The digraph is incorporated in the following word BREED (42). In E333 the left-over tiles generate the array illustrated by Fig. 4: this array leads the participant to suggest the word BONER (E338) then BONED (E342) and finally BONE (E349). This efficient word sequence was sparked by an unplanned change in the array.

### Missed opportunities

**Fig. 5:**
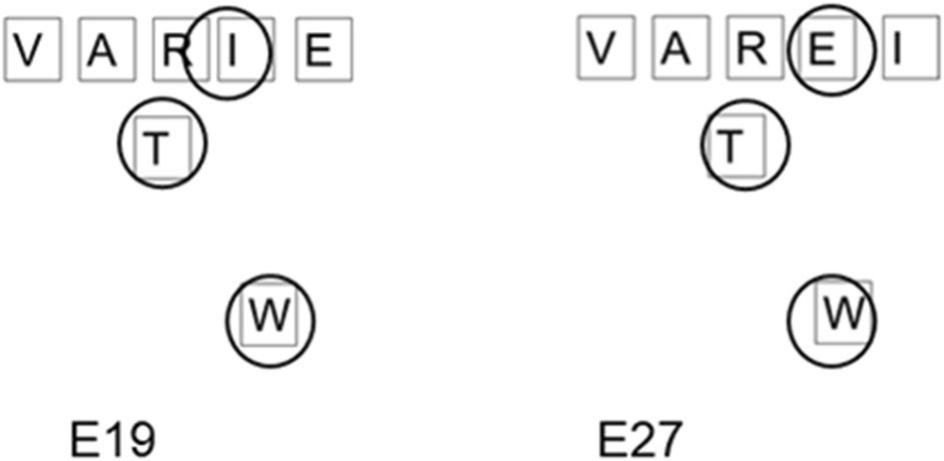
Contrasting WET and **wit** configurations

A serendipitous moment can only arise when the environmental luck is capitalised upon by the problem solver. This is something that P20 found particularly difficult. Her lack of movements meant that fewer lucky arrangements were generated but also, she failed to notice others. For example, E19 and E27 offer almost identical letter array aside from the rearrangement of I and E (see Fig. 5) The word WET is spelt out in a triangle in E27 and is prompted, however, this arrangement has spelt out the less common word **wit** previously but this is not noticed by the problem solver (‘wet’ has a Zipf score of 4.67, ‘wit’ has a Zipf score of 3.67) although being in the same position in E19 as WET is in E27.

**Fig. 6:**
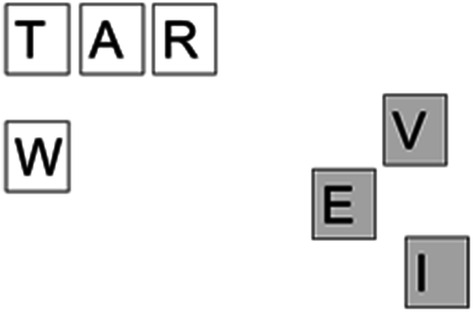
Configurations spelling **tar** which was unrecognised by P20

This underlines the importance of internal and external resources—while the environment may yield a word, if it is not noticed by the problem solver, it will remain inert. This happened often throughout the course of the 5 min for P20. It is particularly noticeable when the participant creates **tar** in E185 but does not say the word. This could be for two reasons, having the word in front of her she forgets to say it (the word does not need to be spoken to take form) or because she simply does not notice it (Fig. 6).

**Fig. 7:**
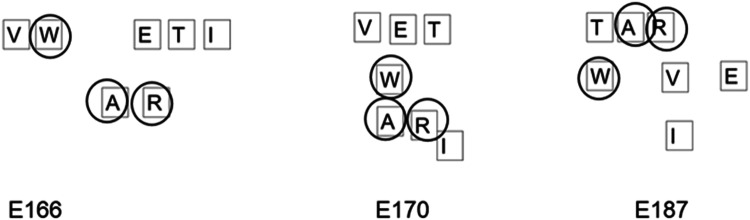
Configurations leading to **war** which were unrecognised by P20

There are other clear moments of P20 not noticing environmental opportunities. In E71–78 the word **ire** is spelt vertically down and yet not recognised by the participant. The word **war** is made several times by the tiles and not recognised. See Fig. 7.

Indeed, any words elicited from the array by this participant were done so in a bottom-up or across way aside from this one last word. Those words which were most obvious and yet missed were those which were spelt downward suggesting an inattentional blindness relating to her habitual gaze trajectory.

**Fig. 8:**
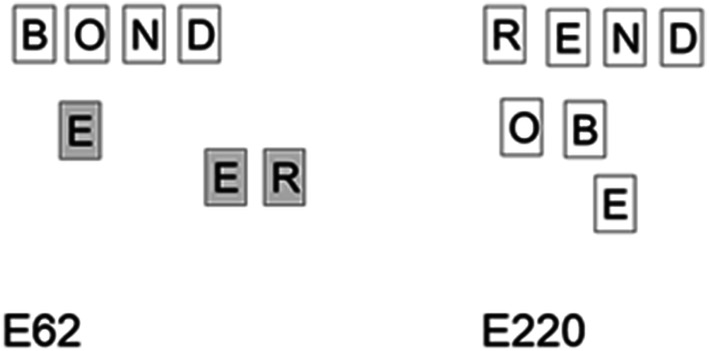
Configurations leading to **bond** and **rend** which were unrecognised by P41

Although we assumed that a high level of engagement would necessarily lead to an improved ability to capitalize on surreptitious candidate words, there are several moments when the array created words which were not noticed by P41 despite his overall high engagement. These were most obvious in the creation of **bond** in E62 and **rend** in E220 the latter of which is not said until E252 as demonstrated in Fig. 8.

This is in part a function of the kinetics of the task environment, which are hard to capture in the static array presented in the appendix. The tiles were in continual movement and the snapshot we have reproduced here cannot capture the fluid landscape of letter combinations. It also reflects a level of disengagement (whether a deliberate strategy or not), a reversed sunk cost as it were, quickly disassembling and re-assembling arrays of letter tiles, which, combined with high verbal fluency, prevents output inertia. Finally, it emphasises the contingent nature of serendipity and the inherent difficulty in observing it. In these instances, the environment yields the luck, the participant has demonstrated the ability to recognise those words, but the moment does not happen.

The additional complexity of a coupled system in movement makes it hard to create a systematic framework for analysing the number of words missed and we present the data here as a starting discussion on how such moments can be categorised. A better understanding of microserendipity will likely involve a better understanding of missed opportunities. However, as a reviewer pointed out, missed opportunities for whom? That is, we identified missed opportunities as we saw them in the video data, but we did not provide a formal way to categorize a certain un-named configuration of letters as a missed opportunity. Informally, horizontal left-to-right series of letters that formed a word and top-to-bottom vertical series of letters that formed a word but which was not named counted as a missed opportunity. However, participants demonstrated a considerable degree of flexibility in the selection and combination of letters to produce words: As illustrated in Fig. 5, P20 produced words in a right-to-left bottom-up manner, and as illustrated in Fig. 4, P41 produced a word in a bottom-top-bottom triangular pattern. This open-ended flexibility complicates considerably the formulation of a priori effort to capture missed opportunities. It seems likely that the missed opportunities will reflect both the characteristics of the system and the individual differences of the problem solver. Inviting participants to watch a video of their performance on the task might be a means to more clearly capture missed opportunities.

## Discussion

Problem solving emerges from a meshwork of dynamic elements. Resources internal and external to the agent configure a cognitive ecosystem that scaffolds performance. In addition, capitalizing on fortuitous external cues may trigger new ideas and these cues in turn may become a constituent part of the cognitive ecosystem. We examined these elements to determine how they come into play during a simple word production task. While the broad aggregate scores of the different conditions showed no significant effect of the experimental manipulation on word production performance, close analysis of the video data led us to conclude that participant behaviour had a large effect on the outcomes: The longer a participant interacted with the tiles, the greater the number of words they produced.

Contrary to expectation, the amount of time spent interacting with the tiles was not related to expertise as measured by the fluency and anagram ability. We expected to see that interactivity was used as a prop by those who required more scaffolding in their problem solving and we also expected that the benefits of interactivity would tail off as people interacted more with the tiles. However, our results suggest a straightforward additive relationship between the time spent interacting with the tiles and the total number of words produced. This suggests that interactivity as a benefit accrues to those who make full use of it and this is regardless of the measured individual differences in verbal fluency and anagram skill. This conclusion was further supported by the significant correlation between efficiency score and performance in the high interactivity condition when such a strategy was easier to enact.

### The benefits of interactivity

Traditional examinations of interactivity have assumed consistencies across participants and across task environments, although the evidence has also indicated that there are significant individual differences in response to an environment that affords interactivity. A research programme dedicated to examining the nature of cognition when coupled to the external world must take seriously both the extent of that coupling and the affordances yielded by different task environments. The change from a digital environment (as in Kirsh, 2014) to the physical problem environment designed in the experiment reported here elicited different behaviours from participants. For example, Participant 41 only chose to shuffle twice in the shuffle condition, but his movements were so quick in the high interactivity condition, with arrays assembled and disassembled swiftly that it seems likely that the behaviour here was much like the behaviour of the participants in the shuffle condition in Kirsh (2014) with the added benefit of being able to interact with the resulting array. A truly systemic model of cognition needs to profile both the individuals and the environment in which they are situated.

There are several possible reasons for the increase in performance that we see when participants make a fuller use of the option to move the tiles. First, the tiles function as external representations which are simply easier to move and enable participants to leverage their own skills. Morphemically probable units can be formed and manipulated without a resultant working memory load. Second, the movements of the tiles may be unplanned, but the rearranging creates liminal and proto words which are exploited by the problem solver. Third, the movements create left-over words which are unanticipated, but then recognised, by the problem solver. The data reported in this paper show a complex interaction of all three. Creative problem solving in a task ecology that favours interacting with the physical elements of a problem is driven by three factors: the internal resources of the problem solver, her embodied behaviour and the environmental affordances that unfold dynamically as the physical model of the problem is modified. A full account of these aspects helps better appreciate their transactional nature.

### The role of luck

Luck is, by its nature, random and when the cost of discarding a shuffle is high, as in the physical environment designed here, the benefits of a lucky shuffle—one which quickly points the problem solver to another words—are outweighed by the high costs of an unlucky shuffle—where the shuffle does not prompt a word in the problem solver. The qualitative case study analysis demonstrated that luck is often a factor in the high interactivity environment as much as in a static random shuffle environment. As our analysis of P41 demonstrates, this fluid task environment allows the participants the possibility of moving the tiles in a unimpeded manner with a much lower time and cognitive cost than the high interactivity version in Kirsh (2014). This supports the need to fully assess how participants are responding to our experimental manipulations.

A framework of microserendipity allows us to make sense of this interaction between the individual and her environment on a micro level. The detailed analysis of behaviour illustrated by our case studies demonstrated a number of missed opportunities across both those who benefitted from interactivity and those that did not. This underlines the relational nature of serendipity. It is only in the act of noticing (Martin & Quan-Haase, 2016) that a serendipitous moment occurs. An environment high in lucky affordances does not guarantee that these will be noticed, but changing the environment often enough to generate these affordances, whether through shuffling on a digital interface or moving in a physical environment, increases the likelihood that they will be realised.

The transactional perspective that informed our analysis of word production performance can also be productively applied to creative problem solving more generally. Much work on so-called insight problem solving proceeds with verbal riddles or static diagrammatic displays of information. This type of second-order problem solving (Vallée-Tourangeau & March, 2019) comes with a hefty representational burden since thinking can only proceed through the mental manipulation of representational states—not unlike the way in which words were produced in the low interactivity condition in the experiment reported here—and hence the psychometric evidence implicating the importance of working memory capacity and general intelligence (e.g., Chuderski & Jastrzębski, 2018) is relatively unsurprising. However, presenting these insight problems in a task environment that allows the manipulation of a physical model of the problem (e.g., Vallée-Tourangeau, 2017; Vallée-Tourangeau et al., 2016) transforms these mental riddles into first-order problem solving, that is thinking with and through the world. As in the high interactivity condition of the word production task reported here, a complex meshwork of cognitive, environmental and serendipitous resources is created as a result. We would argue that such meshwork is much more representative of creative problem solving in the wild. A mixed method analysis strategy, offering aggregate quantitative analysis of performance along with detailed qualitative analyses of the spatio-temporal trajectory of cognition will likely offer a better description of problem solving.

## Footnotes

1 Participants produced the most words in the shuffle condition (*M *= 18.88, SD = 4.14), followed by the interactive condition (*M *= 17.67, SD = 4.31) and then the static condition (*M *= 16.56, SD = 5.35); the main effect of condition was significant, *F*(2,50) = 11.28, *p *< .001. Post-hoc *t* tests revealed that word production performance was significantly higher in the shuffle condition than in the interactive condition, *t *= 2.49, *p *= .02, and significantly higher in the interactive than in the static condition, *t *= 2.20, *p *= .038 (Kirsh, 2014, p. 17).

2 Calculated as the number of letters in the word that were in same absolute position as in the previous word divided by either the number of letters in the word itself or the number of letters in the previous word—whichever was the smallest value—to give a proportion of letters in the same absolute position.

3 Calculated as the number of letters in the same relative position (i.e., follows the same letter as in the previous word) and divided by either the number of letters in the word itself less 1 or the number of letters in the previous word less 1—whichever was the smallest value—to give a proportion of letters in the same relative position.

## References

[CR1] Agnoli S, Franchin L, Rubaltelli E, Corazza GE (2015). An eye-tracking analysis of irrelevance processing as moderator of openness and creative performance. Creativity Research Journal.

[CR2] Beer RD, Frankish K, Ramsey WM (2014). Dynamical systems and embedded cognition. The Cambridge handbook of artificial intelligence.

[CR3] Björneborn L (2017). Three key affordances for serendipity: Toward a framework connecting environmental and personal factors in serendipitous encounters. Journal of Documentation.

[CR4] Bocanegra BR, Poletiek FH, Ftitache B, Clark A (2019). Intelligent problem-solvers externalize cognitive operations. Nature Human Behaviour.

[CR5] Chuderski A, Jastrzębski J (2018). Much ado about aha!: Insight problem solving is strongly related to working memory capacity and reasoning ability. Journal of Experimental Psychology: General.

[CR6] Copeland S (2017). On serendipity in science: Discovery at the intersection of chance and wisdom. Synthese.

[CR7] Destefano M, Lindstedt JK, Gray WD, Carlson L, Holscher C, Shipley T (2011). Use of complementary actions decreases with expertise. Proceedings of the 33rd annual conference of the cognitive science society.

[CR8] Edward Foster A, Ellis D (2014). Serendipity and its study. Journal of Documentation.

[CR9] Fleck JI, Weisberg RW (2013). Insight versus analysis: Evidence for diverse methods in problem solving. Journal of Cognitive Psychology.

[CR10] Fleming, M., & Maglio, P. (2015). How physical interaction helps performance in a Scrabble-like task. In D. C. Noelle, R. Dale, A. S. Warlaumont, J. Yoshimi, T. Matlock, C. D. Jennings, & P. Maglio (Eds.), Proceedings of the 37th annual conference of the cognitive science society (pp. 716–721). Austin, TX: Cognitive Science Society.

[CR11] Friedlander KJ, Fine PA (2016). The grounded expertise components approach in the novel area of cryptic crossword solving. Frontiers in Psychology.

[CR12] Friedlander KJ, Fine PA (2018). “The penny drops”: Investigating insight through the medium of cryptic crosswords. Frontiers in Psychology.

[CR13] Gavurin E (1967). Anagram solving under conditions of letter order randomization. Journal of Psychology.

[CR14] Hutchins E (1995). Cognition in the wild.

[CR15] Kirsh D, Robbins P, Ayeded M (2009). Problem solving and situated cognition. The Cambridge handbook of situated cognition.

[CR16] Kirsh D (2014). The importance of chance and interactivity in creativity. Pragmatics & Cognition.

[CR17] Kirsh D, Maglio P (1994). On distinguishing epistemic from pragmatic action. Cognitive Science.

[CR18] Lee K, Ashton MC (2018). Psychometric properties of the HEXACO-100. Assessment.

[CR19] Maglio PP, Matlock T, Raphaely D, Chernicky B, Kirsh D, Hahn M, Stoness SC (1999). Interactive skill in Scrabble. Proceedings of the 21st conference of the cognitive science society.

[CR20] Makri S, Blandford A (2012). Coming across information serendipitously—Part 1: A process model. Journal of Documentation.

[CR21] Martin K, Quan-Haase A (2016). The role of agency in historians’ experiences of serendipity in physical and digital information environments. Journal of Documentation.

[CR22] McCay-Peet L, Toms EG, Kelloway EK (2015). Examination of relationships among serendipity, the environment, and individual differences. Information Processing and Management.

[CR23] Novick LR, Sherman SJ (2003). On the nature of insight Solutions: Evidence from skill differences in anagram Solution. The Quarterly Journal of Experimental Psychology Section A.

[CR24] Ormerod TC, MacGregor JN, Chronicle EP (2002). Dynamics and constraints in insight problem solving. Journal of Experimental Psychology: Learning, Memory, and Cognition.

[CR25] Pasteur, L. (1854/1922). Oeuvres de Pasteur/réunies par Pasteur Vallery-Radot. Retrieved 29/12/2019 from https://commons.wikimedia.org/wiki/File:Louis_Pasteur_Universit%C3%A9_de_Lille_1854-1857_dans_les_champs_de_l%27observation_le_hasard_ne_favorise_que_les_esprits_pr%C3%A9par%C3%A9s.pdf.

[CR26] Seifert CM, Meyer DE, Davidson N, Patalano AL, Yaniv I, Sternberg R, Davidson J (1994). Demystification of cognitive insight: Opportunistic assimilation and the prepared-mind hypothesis. The nature of insight.

[CR27] Steffensen SV, Vallée-Tourangeau F, Vallée-Tourangeau F (2018). An ecological perspective on insight problem solving. Insight: On theorigins of new ideas.

[CR28] Steffensen SV, Vallée-Tourangeau F, Vallée-Tourangeau G (2016). Cognitive events in a problem-solving task: A qualitative method for investigating interactivity in the 17 Animals problem. Journal of Cognitive Psychology.

[CR29] Thurstone LL (1938). Primary mental abilities.

[CR30] Tuffiash M, Roring RW, Ericsson KA (2007). Expert performance in SCRABBLE: Implications for the study of the structure and acquisition of complex skills. Journal of Experimental Psychology: Applied.

[CR31] Vallée-Tourangeau F (2014). Insight, interactivity and materiality. Pragmatics & Cognition.

[CR32] Vallée-Tourangeau F, Gunzelmann G, Howes A, Tenbrink T, Davelaar EJ (2017). Interactivity and ego depletion in insight problem solving. Proceedings of the 39th annual conference of the cognitive science society.

[CR33] Vallée-Tourangeau F, March PL (2019). Insight out: Making creativity visible. Journal of Creative Behavior.

[CR34] Vallée-Tourangeau F, Steffensen SV, Vallée-Tourangeau G, Sirota M (2016). Insight with hands and things. Acta Psychologica.

[CR35] Vallée-Tourangeau F, Wrightman M (2010). Interactive skills and individual differences in a word production task. AI and Society.

[CR36] van Heuven WJB, Mandera P, Keuleers E, Brysbaert M (2014). Subtlex-UK: A new and improved word frequency database for British English. Quarterly Journal of Experimental Psychology.

[CR37] Webb ME, Little DR, Cropper SJ (2018). Once more with feeling: Normative data for the aha experience in insight and noninsight problems. Behavior Research Methods.

[CR38] Webb S, Vallée-Tourangeau F, Taatgen N, van Rijn H, Nerbonne J, Shomaker L (2009). Interactive word production in dyslexic children. Proceedings of the 31st annual conference of the cognitive science society.

[CR39] Yaqub O (2018). Serendipity: Towards a taxonomy and a theory. Research Policy.

